# Low-dose ipilimumab plus nivolumab combined with IL-2 and hyperthermia in cancer patients with advanced disease: exploratory findings of a case series of 131 stage IV cancers – a retrospective study of a single institution

**DOI:** 10.1007/s00262-020-02751-0

**Published:** 2020-11-05

**Authors:** R. Kleef, R. Nagy, A. Baierl, V. Bacher, H. Bojar, D. L. McKee, R. Moss, N. H. Thoennissen, M. Szász, T. Bakacs

**Affiliations:** 1 Immunology and Integrative Oncology, Auhofstraße 1, 1130 Vienna, Austria; 2 Department of Statistic and Operations Research, University of Vienna, Oskar-Morgenstern-Platz 1, 1090 Vienna, Austria; 3 NextGen Oncology, Dusseldorf, Germany; 4 Integrative Cancer Consulting, Aptos, CA USA; 5 Moss Reports, 104 Main Street, Unit 1422, Blue Hill, ME 04614-1422 USA; 6 Oncology Center at Lenbachplatz, Ottostr. 3, 80333 Munich, Germany; 7 Cancer Centre, Semmelweis University, 1083 Budapest, Hungary; 8 PRET Therapeutics Ltd, 1124 Budapest, Hungary

**Keywords:** Stage IV cancer, Immunotherapy, Hyperthermia, IL-2, Checkpoint inhibitors, irAEs

## Abstract

The 3-year overall survival (OS) rate of patients with previously treated or untreated stage III or IV melanoma has by now reached 63% using ipilimumab and nivolumab therapy. However, immune-related adverse events (irAEs) of grade 3 or 4 occurred in 59% of patients leading to discontinuation of therapy in 24.5% of patients and one death. Therapy with checkpoint inhibitors could be safer and more effective in combination with hyperthermia and fever inducing therapies. We conducted a retrospective analysis to test the safety and efficacy of a new combination immune therapy in 131 unselected stage IV solid cancer patients with 23 different histological types of cancer who exhausted all conventional treatments. Treatment consisted of locoregional- and whole-body hyperthermia, individually dose adapted interleukin 2 (IL-2) combined with low-dose ipilimumab (0.3 mg/kg) plus nivolumab (0.5 mg/kg). The objective response rate (ORR) was 31.3%, progression-free survival (PFS) was 10 months, survival probabilities at 6 months was 86.7% (95% CI, 81.0–92.8%), at 9 months was 73.5% (95% CI, 66.2–81.7%), at 12 months was 66.5% (95% CI, 58.6–75.4%), while at 24 months survival was 36.6% (95% CI:28.2%; 47.3%). irAEs of World Health Organization (WHO) Toxicity Scale grade 1, 2, 3, and 4 were observed in 23.66%, 16.03%, 6.11%, and 2.29% of patients, respectively. Our results suggest that the irAEs profile of the combined treatment is safer than that of the established protocols without compromising efficacy.

**Electronic supplementary material:**

The online version of this article (10.1007/s00262-020-02751-0) contains supplementary material, which is available to authorized users.

## Introduction

Concurrent ipilimumab and nivolumab treatment has by now achieved a 3-year overall survival (OS) rate of 63% for patients with advanced melanoma [1]. However, the treatment-related irAEs were reported in 96.8% of patients, 58.5% of which were grade 3 and 4 leading to discontinuation in 24.5% of patients and one death. Ultimately immune checkpoint therapy (ICI) will continue to increase exponentially, bringing with it a growing burden of irAEs [2]. The meta-analysis of Xing et al. including 48 trials with 7936 patients who were treated with mono-therapeutic nivolumab or nivolumab plus ipilimumab raised the issue that the deleterious effects of severe irAEs might outweigh the benefit from the addition of ipilimumab [3]. Predictive biomarkers might help selecting patients who will derive the greatest benefit from ICIs [4]. However, selection of patients may not be required if lower ICI dosages are used.

### Rationale for low-dose ICI therapy

In the above context it is important to recall that the original rationale for immune checkpoint inhibitor (ICI) therapy was based on the assumption that the blockade selectively targets T-cells relevant to the antitumor immune response [5]. Unfortunately, this assumption cannot be reconciled with the widespread irAEs observed in the vast majority of patients (see for example in [6]). As a matter of fact, Bakacs et al. proposed already in 2012 that the ipilimumab-induced irAEs were very similar to that of a chronic graft-versus-host-disease (GVHD) reaction following *allogeneic* bone marrow transplantation (BMT) [7, 8]. They speculated that ipilimumab induced a graft-versus-malignancy (GVM) effect by the patients’ own lymphocytes, which eradicated metastatic melanoma in a minority of patients, but also involved an auto-GVHD reaction that resulted in widespread autoimmunity in the majority. In the face of an ipilimumab-induced pan-lymphocytic activation, based on an alternative interpretation of the seminal NEJM paper by Hodi et al. [9], a therapeutic paradigm shift was proposed. The task is not desperately trying to put the genie back in the bottle by immune suppressive treatments, but instead harnessing the autoimmune forces for therapeutic purposes. This idea paved the way for administering lower doses of ICI drugs.

Slavin et al. were the first to suggest that a finely tuned, low-dose (0.3 mg/kg) ipilimumab treatment course would induce a prolonged auto-GVHD that would improve the antitumor efficacy of the patients’ own lymphocytes for a broad spectrum of malignancies at the stage of minimal residual disease (MRD) [10]. In this way, the same goal could be achieved by an antibody (ipilimumab) as by the adoptive transfer of alloreactive donor lymphocytes, but of course, without severe GVHD.

**Fig. 1:**
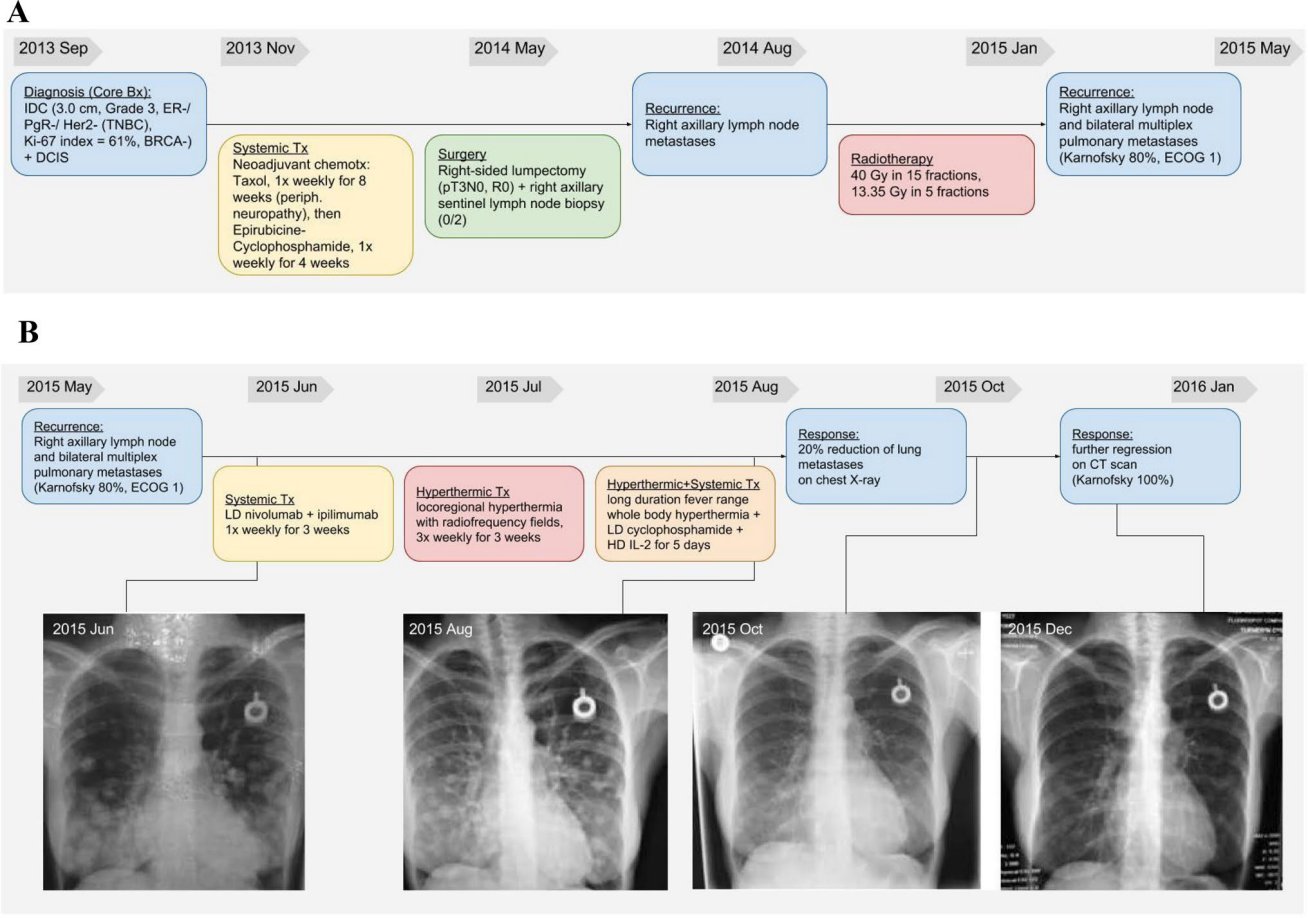
Detailed history and chest X-rays of a patient with triple-negative breast cancer from diagnosis and treatment before (**a**) and after attending (**b**) the outpatient clinic with the respective regimen. Long-term follow-up is also displayed supported by chest X-rays. Reproduced from Kleef et al., Integrative Cancer Therapies 2018, Vol. 17(4) 1297–1303 with permission from SAGE Publishing 2600 Virginia Ave NW, Suite 600 Washington, DC 20,037 USA

The low-dose ICI idea was first adopted by Kleef et al. for stage IV cancer patients [11, 12]. Following the quantitative paradigm of T-cell activation [13, 14], which states that the outcome of signals from the TCR, co-stimulatory/co-inhibitory receptors and cytokines are synergistic, Kleef combined an off label low-dose anti-CTLA-4 plus anti-PD-1 antibody blockade with hyperthermia, and individualized dosing of IL-2 treatment. The synergism of the various T-cell stimulatory effects was first demonstrated in a heavily pre-treated triple negative breast cancer (TNBC) patient, with far advanced pulmonary metastases and severe shortness of breath, who had exhausted all conventional treatment [11]. The patient was treated with a safe, low-dose immune checkpoint blockade, including ipilimumab (0.3 mg/kg) combined with nivolumab (0.5 mg/kg). This was complemented with an individually dosed IL-2 treatment under taurolidine protection and loco regional- and whole-body hyperthermia, without classical chemotherapy. The patient went into complete remission of her lung metastases and all cancer-related symptoms vanished with transient WHO I-II diarrhea and skin rash (Fig. 1a, b). A total gene expression analysis of a metastatic axillary lymph node demonstrated that several checkpoint genes were over-expressed even one year after the initiation of therapy. The patient remained alive for 27 months after the start of treatment, with recurrence of metastases as a sternal mass, and up to 3 cm pleural metastases, which finally classified this patient having a mixed overall response. Following the TNBC patient, the proof-of-principle of this low-dose combination immune checkpoint therapy, consisting only of approved drugs and treatments, was demonstrated in many further cancer patients [12, 15].

A recent model-based meta-analysis that evaluated safety data from 80 published clinical trials (representing 21,305 patients from 153 dosing cohorts) supports the view that combination treatment with CTLA-4 and PD-1 inhibitors increases irAE rates beyond additivity [16]. With the benefit of hindsight, this finding also justifies the rationale of our combined low-dose anti-CTLA-4 plus anti-PD-1 antibody blockade.

## Methods and treatment

All patients signed informed consent to the experimental (off-label) treatment including consent to evaluate patients retrospectively for scientific publication. Unselected patients with 23 cancer types (see patient’s demographics in Table supply 1) were treated in a named patient program with individualized dosing of checkpoint inhibitor therapy, hyperthermia and IL-2. The majority of patients were treated with a combination of anti-program cell death-1 receptor (anti-PD-1) checkpoint inhibitor nivolumab and anti-cytotoxic T-lymphocyte-associated protein-4 (anti-CTLA-4) checkpoint inhibitor ipilimumab with 0.5 mg/kg and 0.3 mg/kg, respectively. The data collection and documentation were retrieved retrospectively from all patients following the completion of therapy. All patient’s data were entered retrospectively into a professional clinical data monitoring system Dendrite^®^ for statistical analysis. Staging was performed with iRECIST in stage IV patients [17]. Checkpoint inhibitor therapy was combined with three modalities: local–regional-, whole-body- and endogenous hyperthermia with IL-2, with the dose titrated individually in each patient to achieve a febrile response of 39–40 °C (temperature was measured by a rectal probe) (Table supply 2).

### Molecular biology diagnosis

It is widely accepted that expression of PD-L1, tumor mutational burden (TMB) and microsatellite instability (MSI) are strongly correlated with positive response towards immunotherapy with checkpoint inhibitors. The overwhelming majority of cancer patients described in the literature have low MSI and TMB with only a small percentage of patient having positive expression of PD-L1. In our patient group, we could not systematically evaluate TMB and MSI, but the majority of our patients have undergone next generation sequencing (NGS) analysis on tumor biopsies, circulating tumor cells (CTC) assays and tumor chemosensitivity assays (TCA). The immunohistochemical determination of PD-L1 was performed by PD-L1 IHC 22C3 pharmDx (Dako North America, Inc) (data not shown).

### Patients demographics

The baseline patient and disease characteristics are presented in Table supply 1.

### Treatment

All patients received the combination treatment described below, if not stated otherwise.

**Fig. 2:**
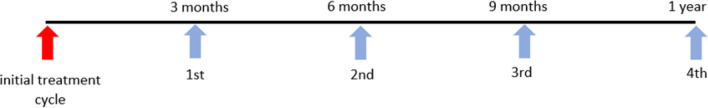
Treatment courses were repeated every 3 months until reaching 3–4 cycles in total in the absence of disease progression or unacceptable toxicity

1. Nivolumab was administered IV over 60 min on days 1, 15, and 29 and ipilimumab IV over 90 min on day 1 and day 15. Courses were repeated every 3-month in the absence of disease progression or unacceptable toxicity (Fig. 2).

2) Loco-regional hyperthermia (radiofrequency device 13.56 MHz, Syncrotherm/ Oncotherm) 3×/weekly 60′ over 4 weeks over the tumor area, 12 txts [18, 19].

3) Long duration whole body hyperthermia (w-IRA Heckel HT3000) under light sedation one time in the week prior to IL-2 therapy with moderate dosed cyclophosphamide 300 mg/m^2^ to down-modulate expected upregulation of Treg cells [20, 21]. Treg cell numbers (available in 37 patients) was indeed down-modulated to 28.1% during therapy and then upregulated to 107.1% after therapy; paired *t* tests for all three comparisons (before vs. during, during vs. after, before vs. after) were significant with *p* values < 0.001 (Table 2).

4) IL-2 (Proleukin^®^) – induced fever therapy: 1 × /month daily 4 × over 1-week outpatient treatment with individually dose adapted IL-2 and Taurolidine. The addition of Taurolidine is mitigating the well-known cytokine storm induced by IL-2, and thus increasing the safety of our protocol [22]. Specifically, Taurolidine was used both during ICI infusions and IL-2 treatment; during ICI treatment patients received 3 × 250 ml of 2% Taurolidine; the dosage during IL-2 treatment was 2 × 250 ml Taurolidine 2% over 8 h. IL-2 was titrated to daily fever responses of 39.0–40 °C depending on the clinical condition. IL-2 was applied via motor-syringe pump with a total dosage of 5–14 Mio/m^2^ and infusion speed of 5–7.5 ml/h/day depending on the clinical condition and fever induction response; depending on the fever response the infusion was stopped; IL-2 was perfused to a maximum fever of 38.5 °C; still, the fever curve always was expected to rise up to a temperature level of 39–40 °C body core temperature measured with rectal probe. Monitoring was performed with continuous measuring of body core temperature, blood pressure, heart rate and oxygen saturation SpO_2_ (Mindray® bio-monitor) as well as daily routine laboratory assessments.

Courses were repeated every 3 months until reaching three cycles in total in the absence of disease progression or unacceptable toxicity. Additional chemotherapy, targeted therapy and hormonal therapy was allowed and documented meticulously (Table supply 2).

Parallel to local regional hyperthermia patients received 3 times weekly high dose vitamin C intravenously (0.5 g/kg) and alpha lipoic acid 600 mg. High-dose vitamin C has been suggested as an adjuvant cancer treatment as it is toxic to tumor cells, since it targets many of the mechanisms that cancer cells utilize for their survival and growth. High-dose vitamin C delays cancer growth by enhancing infiltration of the tumor microenvironment by immune cells and cooperates with immune checkpoint therapy (ICT) in several cancer types [23–26]. Lipoic acid synergistically enhanced ascorbate cytotoxicity [27] and inhibited the enzyme pyruvate dehydrogenase kinase that is particularly upregulated in cancer cells and is the major determinant of the “Warburg effect”, thus contributing to a higher vulnerability/decreased resilience of the neoplasm [28].

### Statistical analysis

OS and PFS were defined as the time from admission until the patients’ death and the first evidence of disease progression, respectively. All continuous variables are presented by median and first and third quartile. Absolute and relative frequencies were derived for categorical data. Median follow-up for OS was estimated by reverse Kaplan–Meier method. Kaplan–Meier curves including 95% confidence intervals were derived for OS and PFS. Cox proportional hazard models for OS and PFS were estimated with diagnosis “breast cancer” as explanatory dummy variable. Differences in Treg cell numbers before, during, and after therapy were analyzed using paired *t* tests. 95% confidence intervals were estimated for outcomes, including dichotomous measures and median survival times. All statistical analyses were performed with the statistical software R version 3.6.1 (R Core Team 2019).^1^

## Results

**Fig. 3:**
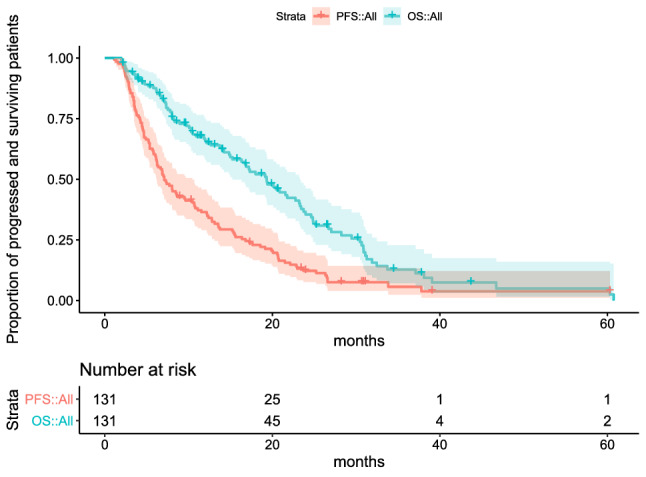
Kaplan–Meier curves of progression-free survival (PFS) and overall survival (OS) in all the 131 cancer patients. 95% confidence intervals are indicated by filled areas around curves, censored observations by “ + ”

**Fig. 4:**
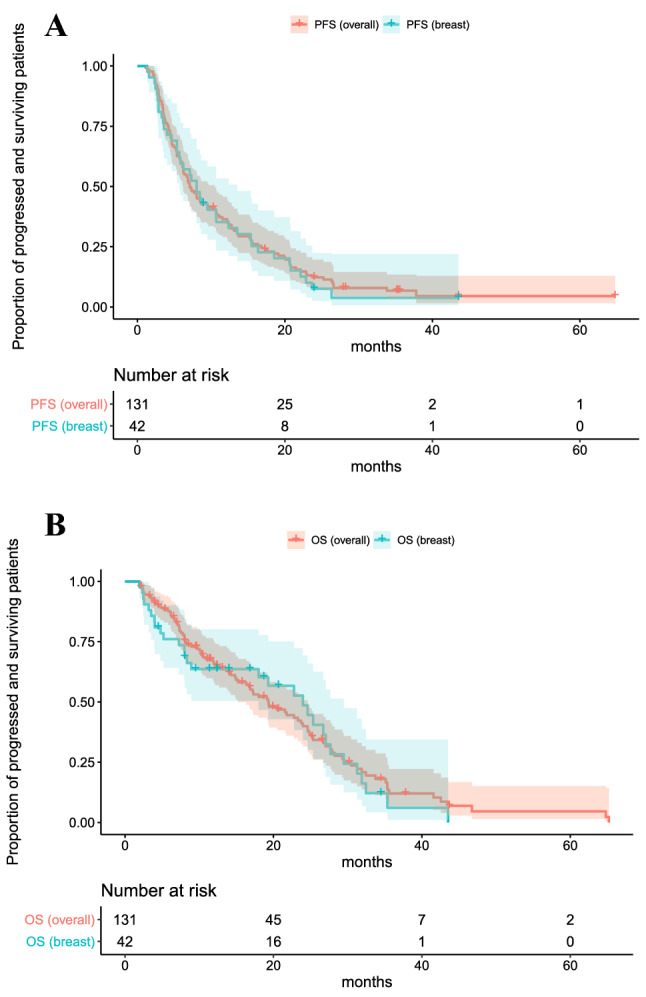
Comparisons of Kaplan–Meier curves of PFS (**a**) and OS (**b**) in all the 131 cancer patients and in 42 breast cancer patients. The hazard ratio (HR) for the difference between all the 131 cancers and 42 breast cancers was estimated using a Cox proportional hazard model and no significant differences were found (PFS: HR: 0.918, 95% CI: [0.742, 1.600] *P*: 0.661; OS: HR: 0.901, 95% CI: [0.715, 1.722] *P*: 0.643)

Stage IV cancer patients (staging with iRECIST; *n* = 131) demonstrated objective response rate (ORR) 31.3%, overall response rate (OR) 49.62%: complete response (CR) 20 patients, partial response (PR) 21 patients, no change (NC) 24 patients, stable disease (SD) 65 patients, and mixed response (MR) 1 patient, progression-free survival (PFS) was 10 months. Survival probabilities at 6, 9, 12, 24 months, irAEs of WHO grading and outcome measures are presented in Tables 1 and 2. Kaplan–Meier curves of PFS and OS in all the 131 cancer patients are presented in Fig. 3. Comparisons of Kaplan–Meier curves of PFS and OS in all the 131 cancer patients and in 42 breast cancer patients are presented in Fig. 4a, b, respectively. Hazard ratios (HR) for the difference between all cancers and breast cancers were not significant (OS: HR: 0.901, 95% CI: [0.715, 1.722] *P*: 0.643; PFS HR: 0.918, 95% CI: [0.742, 1.600] *P*: 0.661).

**Table 1:** Survival probabilities and irAEs

Survival-probabilities at	
6 months	87.6% [95% CI: 82%; 93.5%]
9 months	72.9% [95% CI: 65.5%; 81.1%]
12 months	65.9% [95% CI: 58%; 74.9%]
24 months	36.6% [95% CI: 28.2%; 47.3%]
irAEs of WHO	
Grade 1	23.66%
Grade 2	16.03%
Grade 3	6.11%
Grade 4	2.29%

## Discussion

Here, we conducted a retrospective analysis of 131 unselected stage IV cancer patients with 23 different cancer types who were treated with T-cell-stimulating modalities, hyperthermia, IL-2, and ipilimumab plus nivolumab (0.5 mg/kg and 0.3 mg/kg, respectively). Twenty-four percent of our patients had to receive antibiotics. Antibiotic treatments administered within 30 days from commencement of ICI therapy has been known to be associated with significantly worse overall survival in patients with nonsmall cell lung cancer (2.5 vs. 26 months), melanoma (3.9 vs. 14 months), and other tumor types (1.1 vs. 11) who received antibiotic treatment vs. those who did not. In addition, patients had a higher risk of disease refractory to treatment [29].

**Table 2:** Outcome measures

Variable	Value (95% CI)
ORR	
Overall	31.3% [24.0%; 39.7%]
With antibiotics treatment	26.5% [14.6%; 43.1%]
Primary tumor location: Breast	31.0% [19.1%; 46.0%]
Primary tumor location: Colon	27,3% [9,7; 56,6%]
Primary tumor location: Ovary	36,4% [15,2; 64,6%]
Primary tumor location: Prostate	45,5% [21,3; 72%]
Checkpoint Inhibitor*: Ipilimumab, Nivolumab	32,2% [24,3; 41,2%]
Checkpoint Inhibitor*: Nivolumab	23,1% [8,2; 50,3%]
*only “ipi + nivo” und “nivo” are included as *n* for other combinations are too low	
ECOG 2 or 3	23.3% [11.8%; 40.9%]
Overall survival	
6 months	87.6% [82%; 93.5%]
9 months	72.9% [65.5%; 81.1%]
12 monts	65.9% [58%; 74.9%]
Median (months)	19.3 [15.2; 22.9]
Progression-free survival	
6 months	60.3% [52.5%; 69.3%]
9 months	42.7% [35.1%; 52.1%]
12 monts	36.4% [29%; 45.7%]
Median (months)	7.1 [6.2; 10.5]
20 patients (15.3%) had CR; their median time until progression was 20.7 months	
Median time until progression for subgroups (all diagnosis with *n* > 10)	
Breast	8.0 months
Colon	6.4 months
Ovary	4.5 months
Prostate	12.8 months
Others	7.5 months
Treg cell numbers*	
Before therapy	60,4% [49,6; 71,1%]
During therapy	28,1% [21,9; 34,3%]
After therapy	107,1% [84,3; 129,9%]

*At least one measurement was available for 37 patients; paired *t* tests for all three comparisons (before vs. during, during vs. after, before vs. after) were significant with *P* values < 0.001

During a follow-up period of up to 5 years, the ORR and OR were 31.3% and 49.62%, respectively, while the OS at 12 months was 65.9% (Table 2). irAEs of WHO grade 1, 2, 3, and 4 were observed in 23.66%, 16.03%, 6.11%, and 2.29% of patients, respectively (Table 3). In other words, less than half (48.09%) of the patients experienced irAEs of any grade, while only 8.4% had grade 3 or 4 irAEs, but no treatment-related death occurred. Therefore, our protocol does not require selection of stage IV cancer patients who are more likely to gain benefit from ICIs. Our safety results compare favorably to that of the study by Callahan et al. but also to the meta-analysis of Xu et al., in both of which registered doses of concurrent nivolumab and ipilimumab treatment were administered. Any-grade irAEs occurred in 96.8% of patients in Callahan’s study, while 58.5% of patients had grade 3 and 4 irAEs leading to discontinuation in 24.5% of patients, and one treatment-related death was also reported [1]. Similarly, grade 3–4 irAEs occurred in 39.9% of patients in the meta-analysis of Xu et al., while 2.0% treatment-related death was recorded [30].

**Table 3:** Immune-related adverse events

Group	*N* (%)
Grade	
1	31 (23.7%)
2	21 (16%)
3	8 (6.1%)
4	3 (2.3%)
No irAE	68 (51.9%)
irAE (Grade I)	
Diarrhea	12 (9.2%)
Skin rash	11 (8.4%)
Mildly elevated GOT/GPT	3 (2.3%)
Pruritus	3 (2.3%)
Dyspnea	2 (1.5%)
Mouth ulcers	2 (1.5%)
Nausea	2 (1.5%)
Thyroid dysfuction	2 (1.5%)
Abdominal dyscomfort	1 (0.8%)
Dry cough	1 (0.8%)
Elevated blood Glc	1 (0.8%)
Flu-like symptoms	1 (0.8%)
Melena	1 (0.8%)
irAE (Grade II)	
Moderately elevated GOT/GPT	10 (7.6%)
Mild pneumonitis	7 (5.3%)
Massive diarrhea	1 (0.8%)
Massive edema	1 (0.8%)
Quincke-edema	1 (0.8%)
Strong skin rash	1 (0.8%)
irAE (Grade III)	
Autoimmune hepatitis (3)	3 (2.3%)
Autoimmune thyroiditis (3)	3 (2.3%)
Autoimmune colitis (3)	2 (1.5%)
irAE (Grade IV)	
Autoimmune DM (4)	2 (1.5%)
Acute kidney injury (4)	1 (0.8%)
Autoimmune thyroiditis (4)	1 (0.8%)

The combination of ipilimumab and nivolumab has first been established in melanoma [31] and renal cell cancer [32] but also described for nonsmall cell lung cancer [33, 34]. In these patients, it is well known that high mutational burden favors better response rates [35]. Although immunotherapy has become one of the greatest advances in oncology over the last century, the application for the treatment of breast cancer remains an area of investigation [36]. Consistent with this, we found 1365 papers with the key words < cancer, ipilimumab, nivolumab > but only 18 with the key words < breast cancer, ipilimumab, nivolumab > (PubMed search as of June 2020). Based on our data presented in this paper, such neglect of breast cancer seems to be unjustified. We estimated the hazard ratio (HR) for the difference between all the 131 cancers and 42 breast cancers using a Cox proportional hazard model and found no significant differences (Fig. 4a, b). 95% confidence intervals for HRs provide ranges of plausible values for the true HRs of our data.

Importantly, the overwhelming majority (> 98%) of the evaluated patients in this study had low PD-L1 expression as confirmed by immunohistochemistry (determined as ≤ 1%). Low tumor mutational burden (TMB^low^) and low microsatellite instability (MSI^low^) was determined only in a small subgroup of patients. However, TMB^low^ and MSI^low^ can be expected in the majority based on published evidence [37]. In contrast, published meta-analyses included patients with high PD-L1 expression, TMB^high^, and MSI^high^ [30, 38]. Therefore, in contrast to the published meta-analysis, our patient group had the following negative pre-selection factors:

- Antibiotic use in 24%;
- Low PD-1/PD-L1 expression;
- Only stage IV patients, with 35.1% liver metastasis, a specifically bad prognosis;
- Only 35% ECOG 0;
- Heavily pretreated patients.

Control of minimal residual cancer by exploiting autoimmunity induced by low-dose ipilimumab was proposed earlier [10]. For safety reasons, we administered the lowest doses of nivolumab (0.5 mg/kg) and ipilimumab (0.3 mg/kg), which induced grade 3 or 4 irAEs in only 8.4% of patients (Table 1.). The low dose ICI protocol was justified by Sen et al., who demonstrated that despite a dose-dependent increase in irAEs, no improvement in PFS, OS, or disease control rate (DCR) were identified with escalating doses of ICIs [39]. The authors concluded that lower doses may reduce toxicity and cost without compromising disease control or survival. In fact, due to the rapidity of development, competition, and race for FDA approval, the optimal dosing and schedule of ICIs are still not fully defined and continue to be under study [40].

Weber predicted 10 years ago that abrogation of the CTLA-4 function results in immune stimulation, tolerance breakdown and eventually tumor eradication [41]. This prediction has recently been formally confirmed by Eggermont et al. in a study including 1019 adults with stage III melanoma, in which patients were randomly assigned on a 1:1 ratio to receive treatment with pembrolizumab therapy or placebo [42]. The occurrence of an irAE was indeed associated with a significantly longer recurrence-free survival (RFS) in the pembrolizumab arm.

ICIs are more likely to halt tumor growth in patients with a higher TMB than in those with a lower one [43, 44]. According to the consensus of experts, mutational load in cancer cells (e.g., lung cancer patients with former nicotine abuse) may generate novel antigens that are not subject to immune tolerance and allow for an *adaptive* immune response by the host. We proposed an alternative interpretation for the induction of immune response. Tumor cells with newly expressed neoantigens are no longer recognized as “self” because they are transformed into “non-self” such that they become targets for the patient’s own immune system. Owing to the newly expressed neoantigens the unresponsiveness/tolerance that existed between the patient’s immune system and cancer cells was abolished. This in turn, resulted in the development of an auto-GVHD with secondary therapeutic benefits, in analogy with the GVM effects following allogeneic stem cell transplantation [15]. Although a limited transformation is too weak in itself to instigate a tumor eradicating T-cell attack, with immune checkpoint blockade T cells are more effective against “altered self” resulting in better OS [45]. Not unexpectedly, a significant positive correlation was found between the reporting odds ratio (ROR) of reporting an irAE during anti-CTLA-4, anti-PD-1, and anti-CTLA-4/anti-PD-1 immune combination therapies and the corresponding TMB in 7677 patients across 19 cancer types [46]. Consistent with this, Berner et al. demonstrated in NSCLC that T cells recognize and target shared tumor and skin antigens during ICI therapy resulting in autoimmune-mediated skin toxicity and tumor regression [47].

### Fever therapy and checkpoint inhibitors

Combining thermal therapy with immune therapy should greatly increase the response rate of immune therapy as all types of hyperthermia appear to increase the numbers of effector lymphocytes [48, 49]. Inducing one week of daily cyclic high fever response during individually dose adapted IL-2 we are in the footsteps of William B. Coley, the father of cancer immunotherapy who administered an FDA-approved fever inducing bacterial inoculate for the treatment of soft tissue sarcomas [50]. It is worth to recall that in 1891 Coley was convinced that stimulating the immune system by a severe infection (such as erysipelas), which was associated with high fever, would have the side effect of shrinking the malignant tumor [51]. By the end of his career, Coley had written over 150 papers and treated almost 1000 cases and noticed that in 500 of these there was near-complete regression [52].

Hyperthermia is almost always used in combination with other forms of cancer therapy [49, 53]. While many studies have shown a significant reduction in tumor size, just a few could demonstrate a moderately increased survival in patients receiving the combined treatments (e.g., [19]). Our response rates in stage IV patients with unfavorable MSI^low^, PD-L1 < 1%, TMB^low^, 26% of which received antibiotics are promising. Since the proposed protocol consists only of approved drugs and treatments, our hypothesis that low‐dose ICI‐induced autoimmune T cells are powerful therapeutic tools can be confirmed or refuted in controlled prospective clinical trials.

### Limitations

A retrospective analysis of case series cannot replace a controlled prospective clinical trial, the gold standard for the evaluation of new treatments. Furthermore, this study was underpowered to detect differences between survival rates in cancer subgroups. Notwithstanding, case reports and series represent relevant study designs, which can be highly influential in furthering medical knowledge despite of their limitations when a question of importance cannot be addressed by other methods because of ethical or logistical constraints [54, 55].

## Conclusions

Here we demonstrated in 131 unselected stage IV cancer patients that hyperthermia, combined with individually dose adapted IL-2 treatment and low doses of nivolumab plus ipilimumab, can be converted from a palliative therapy into a treatment with curative intent because the autoimmune forces unleashed by ICI drugs can be harnessed by a multicomponent T-cell stimulation therapy. It is tempting to speculate whether adding an oncolytic virus (e.g., the oncolytic Newcastle Disease Virus^2^) or novel bacterial vaccines [56] to the protocol would break therapy resistance of patients who respond poorly to this new immune therapy [57, 58].

### Electronic supplementary material

Below is the link to the electronic supplementary material.

Supplementary file1 (DOCX 36 kb)

## Abbreviations

auto-GVHD: Autologous-graft-versus-host-like-disease
CTLA-4: Cytotoxic T-lymphocyte-associated protein-4
GVM: Graft-versus-malignancy effect
ICI: Immune checkpoint inhibitors
irAEs: Immune related adverse effects
IL-2: Interleukin-2
MSI^low^: Low microsatellite instability
NK cell: Natural killer cell
ORR: Objective response rate
OR: Overall response rate
OS: Overall survival
PD-1: Programmed cell death-1 protein
PFS: Progression free survival
RECIST: Response Evaluation Criteria in Solid Tumors
TMB^low^: Low tumor mutation burden
WBH: Whole-body hyperthermia

## Footnotes

1 https://www.eea.europa.eu/data-and-maps/indicators/oxygen-consuming-substances-in-rivers/r-development-core-team-2006

2 https://stm.sciencemag.org/content/10/422/eaam7577/tab-e-letters
