# Supplementary material for: Low-dose ipilimumab plus nivolumab combined with IL-2 and hyperthermia in cancer patients with advanced disease: exploratory findings of a case series of 131 stage IV cancers – a retrospective study of a single institution
Source: Cancer Immunol Immunother. 2020 Nov 5;70(5):1393–403. doi: 10.1007/s00262-020-02751-0 (PMC8053148; doi:10.1007/s00262-020-02751-0)
Supplement: Supplementary file 1 — Supplementary file1 (DOCX 36 kb) [file 262_2020_2751_MOESM1_ESM.docx]

**TABLE 1 : Baseline Patient and Disease Characteristics**

| Characteristic | N = 131 |
| --- | --- |
| Male sex | 47 (35.9%) |
| Age | 57 (49-65) |
| UICC Stage IV | 131 (100%) |
| ECOG |  |
| - 0 | 40 (30.5%) |
| - 1 | 61 (46.6%) |
| - 2 | 26 (19.8%) |
| - 3 | 4 (3.1%) |
| Time from first diagnosis to admission (months) | 26.0 (11.8-50.1) |
| Lymphocyte counts | 1263 (844-1717) |
| Neutrophil counts | 3936 (2709-4924) |
| LDH | 211 (182-279) |
| Palete counts | 236 (201-290) |
| Systemic Immune-Inflammation Index (SII) | 732 (426-1270) |
| Primary tumor location |  |
| - Breast/TNBC | 42 (32.1%)/12(26,8%) |
| - Colon | 11 (12.4%) |
| - Ovary | 11 (13.8%) |
| - Prostate | 11 (13.8%) |
| - Pancreas | 8 (10.7%) |
| - Bronchus and lung | 8 (7.1%) |
| - Stomach | 4 (2.4%) |
| - Kidney, except renal pelvis | 4 (2.4%) |
| - Bone and articular cartilage of limbs | 3 (1.8%) |
| - Skin | 3 (1.6%) |
| - Mesothelioma | 3 (1.6%) |
| - Retroperitoneum and peritoneum | 3 (1.3%) |
| - Other connective and soft tissue | 3 (1.3%) |
| - corpus uteri | 3 (1%) |
| - Bladder | 3 (1%) |
| - Esophagus | 2 (0.7%) |
| - cervix uteri | 2 (0.6%) |
| - Base of tongue | 1 (0.3%) |
| - Liver and intrahepatic bile ducts | 1 (0.3%) |
| - Gallbladder | 1 (0.3%) |
| - Other and unspecified parts of biliary tract | 1 (0.3%) |
| - Thymus | 1 (0.3%) |
| - Testis | 1 (0.3%) |
| - Non-Hodgkin lymphoma | 1 (0.3%) |
| Site of metastases |  |
| - Lymphatic (lymph node involvement) | 65 (49.6%) |
| - Skeletal | 55 (42.0%) |
| - Hepatic | 46 (35.1%) |
| - Pulmonal | 45 (34.4%) |
| - Liver | 41 (31.3%) |
| - Peritoneal | 24 (18.3%) |
| - Cerebral | 11 (8.4%) |
| - Pleural | 7 (5.3%) |
| - Cutaneous | 6 (4.6%) |
| - Renal | 4 (3.1%) |
| - Splenic | 3 (2.3%) |
| - Adrenal | 2 (1.5%) |

Data presented as N (%) for categorial variables and median (Q1-Q3) for continuous variables

**TABLE 2: Treatment Regimes**

| Treatment type | N (%) |
| --- | --- |
| Antibiotics use | 34 (26%) |
| Checkpoint Inhibitor |  |
| - Ipilimumab, Nivolumab | 115 (87.8%) |
| - Nivolumab | 13 (9.9%) |
| - Ipilimumab,Nivolumab,Pembrolizumab | 2 (1.5%) |
| - Ipilimumab | 1 (0.8%) |
| Checkpoint Inhibitor - cycles |  |
| - 1 | 9 (6.9%) |
| - 2 | 17 (13.0%) |
| - 3 | 91 (69.5%) |
| - 4 or more | 14 (10.7%) |
| Chemotherapy - number of treatments |  |
| - 0 | 71 (54.2%) |
| - 1 | 22 (16.8%) |
| - 2 | 16 (12.2%) |
| - 3 | 11 (8.4%) |
| - 4 or more | 11 (8.4%) |
| Chemotherapy - treatment types* |  |
| - Gemcitabine | 27 (20.6%) |
| - Paclitaxel | 16 (12.2%) |
| - Docetaxel | 9 (6.9%) |
| - Nab-Paclitaxel | 3 (2.3%) |
| - Oxaliplatin | 6 (4.6%) |
| - Hyperthermia treatment | 131 (100%) |
| IL-2 - number of treatments |  |
| - 1 | 122 (93.1%) |
| - 2-4 | 9 (6.9%) |
| Radiation | 4 (3.1%) |
| Infusion Therapy | 128 (97.7%) |

* Number of patients that received treatment type at least once. Treatment types with less than 10 occurrences are not listed.
